# From *Ficus recemosa* Leaf Galls to Therapeutic Silver Nanoparticles: Antibacterial and Anticancer Applications

**DOI:** 10.3390/pharmaceutics16081025

**Published:** 2024-08-01

**Authors:** Ovungal Sabira, Nedumbayil Drisya, Anthyalam Parambil Ajaykumar, Asok Mathew, Kodangattil Narayanan Jayaraj, Valiyaparambil Sivadasan Binitha, Koladath Vasu Zeena, Kanakkassery Balan Roy, Pandikkadan Ayyappan Janish, Padannappurath Sheena, Kaladharan Perumpaparampil Viswanathan

**Affiliations:** 1 Division of Biomaterial Sciences, Department of Zoology, Sree Neelakanta Government Sanskrit College, Pattambi 679303, Kerala, India; sabirao@sngscollege.org (O.S.); drisyanv2001@gmail.com (N.D.); zeenasalim@gmail.com (K.V.Z.); janishpa@sngscollege.org (P.A.J.);; 2 Clinical Sciences Department, Centre for Medical and Bio-Allied Health Sciences Research, Ajman University, Ajman P.O. Box 346, United Arab Emirates; a.mathew@ajman.ac.ae; 3 Basic Sciences Department, Centre for Medical and Bio-Allied Health Sciences Research, Ajman University, Ajman P.O. Box 346, United Arab Emirates; 4 Department of Zoology, Sree Narayana College, Nattika 680566, Kerala, India; binisncn@gmail.com; 5 Department of Chemistry, Sree Neelakanta Government Sanskrit College, Pattambi 679303, Kerala, India; roykb@sngscollege.org; 6 Department of Statistics, Maharajas College, Kochi 682011, Kerala, India; kaladharan@maharajas.ac.in

**Keywords:** *Ficus recemosa*, silver nanoparticles, LC-MS Q-TOF, trypan blue assay, antibacterial activity

## Abstract

The synthesis of silver nanoparticles (AgNPs) using environmentally friendly methods has become increasingly important due to its sustainability and cost-effectiveness. This study investigates the green synthesis of AgNPs using gall extracts from the plant *Ficus recemosa*, known for its high phytochemical content. The formation of AgNPs was verified through multiple analytical techniques, including UV-Vis spectroscopy, Fourier-transform infrared (FTIR) spectroscopy, transmission electron microscopy (TEM), zeta potential analysis, and dynamic light scattering (DLS). The UV-Vis spectroscopy results displayed a distinct surface plasmon resonance peak indicative of AgNP formation. FTIR analysis revealed specific interactions between silver ions and phytochemicals in the gall extract, while TEM images confirmed the nanoscale morphology and size of the synthesized particles. Zeta potential and DLS analyses provided insights into the stability and size distribution of the AgNPs, demonstrating good colloidal stability. Biological properties of the AgNPs were assessed through various assays. Antimicrobial activity was tested using the disc diffusion method against *Escherichia coli* and *Staphylococcus aureus*, showing significant inhibitory effects. The anticancer potential was evaluated using the trypan blue exclusion assay on Dalton’s Lymphoma Ascites (DLA) cells, revealing considerable cytotoxicity. Additionally, antimitotic activity was studied in the dividing root cells of *Allium cepa*, where the AgNPs significantly inhibited cell division. This research highlights the effective use of *F. recemosa* gall extracts for the green synthesis of AgNPs, presenting an eco-friendly approach to producing nanoparticles with strong antimicrobial, anticancer, and antimitotic properties. The promising results suggest potential applications of these biogenic AgNPs in medical and agricultural sectors, paving the way for further exploration and utilization.

## 1. Introduction

The synthesis of nanoparticles has recently drawn considerable scientific interest due to their distinctive physical, chemical, and biological properties, which markedly differ from their bulk material counterparts [1,2,3]. Among the various nanoparticles, silver nanoparticles (AgNPs) are particularly notable for their remarkable antimicrobial, anti-inflammatory, and catalytic properties [4,5,6,7]. Traditional methods for synthesizing AgNPs often involve chemical and physical processes that are energy-intensive and environmentally harmful due to the use of toxic chemicals. As a result, there is a growing interest in developing environmentally friendly and sustainable methods for nanoparticle synthesis, particularly through biological means. Biological synthesis, or “green synthesis”, of nanoparticles uses natural reducing agents found in biological entities such as plants, bacteria, fungi, and algae [8,9,10]. Plant-mediated synthesis is particularly attractive due to its simplicity, cost-effectiveness, and potential for large-scale production. Plants are rich in various phytochemicals, including polyphenols, flavonoids, and alkaloids, which can act as reducing and capping agents during nanoparticle formation. This approach not only reduces the need for harmful chemicals but also enhances the biocompatibility of the synthesized nanoparticles [8,9,11].

When plants are affected by nematodes, bacteria, fungi, insects, or mites, they can develop abnormal vegetative growths known as plant galls. These galls result from a close interaction between the gall-inducing organism and the host plant. While galls can form on any part of the plant, they are most commonly observed as large, swollen growths on leaves, petioles, twigs, or branches [12]. Galls on plant leaves or twigs are generally considered harmful to the host. However, they can also benefit the plants by attracting frugivores, which play a crucial role in seed dispersal [13]. Previous research has shown the production of silver nanoparticles (AgNPs) from plant galls, specifically those from *Quercus infectoria* and *Rhus chinensis* [14]. Aqueous extracts from *Q. infectoria* galls have been used to produce AgNPs quickly, cost-effectively, and efficiently, serving as both reducing and capping agents [15]. Similarly, a green synthesis method has been used to create AgNPs from silver nitrate solutions using water extracts from *R. chinensis* galls as reducing agents [14]. Plant galls are used for nanoparticle preparation due to their numerous advantageous qualities. They are rich in phytochemicals, such as flavonoids, tannins, and phenolics, which act as stabilizing and reducing agents in nanoparticle formation [16]. This sustainable and green approach avoids harmful chemicals, thereby reducing environmental impact. Additionally, plant galls, often considered waste, provide a cost-effective method for nanoparticle production. Nanoparticles synthesized from plant extracts generally exhibit improved properties, including stability, biocompatibility, and efficacy in various applications such as environmental remediation, agriculture, and medicine. These benefits make plant galls a valuable resource in nanotechnology.

This study focuses on the green synthesis of silver nanoparticles using the gall part of the plant *Ficus recemosa*, known for its medicinal properties, which belongs to the *Moraceae* family and is native to various tropical regions [17,18]. The galls of *F. recemosa* are particularly rich in phytochemicals such as tannins, saponins, and flavonoids, which are hypothesized to play a crucial role in the reduction of silver ions (Ag+) to silver nanoparticles (AgNPs) and in stabilizing the nanoparticles [19,20]. By utilizing the galls of *F. recemosa*, this research aims to contribute to the expanding field of green nanotechnology and provide insights into the synthesis mechanism, characterization, and potential applications of biogenic silver nanoparticles. The methodology employed involves the extraction of phytochemicals from *F. recemosa* galls and their subsequent use in the synthesis of AgNPs. The synthesized nanoparticles will be characterized using various analytical techniques, including UV-Vis spectroscopy and high-resolution transmission electron microscopy (HR-TEM), to determine their size, shape, and nature. Additionally, the antimicrobial efficacy, anticancer efficacy, and antimitotic properties of the AgNPs was evaluated, demonstrating their potential utility in medical and environmental applications.

## 2. Methodology

### 2.1. Collection of Plant Material

**Figure 1:**
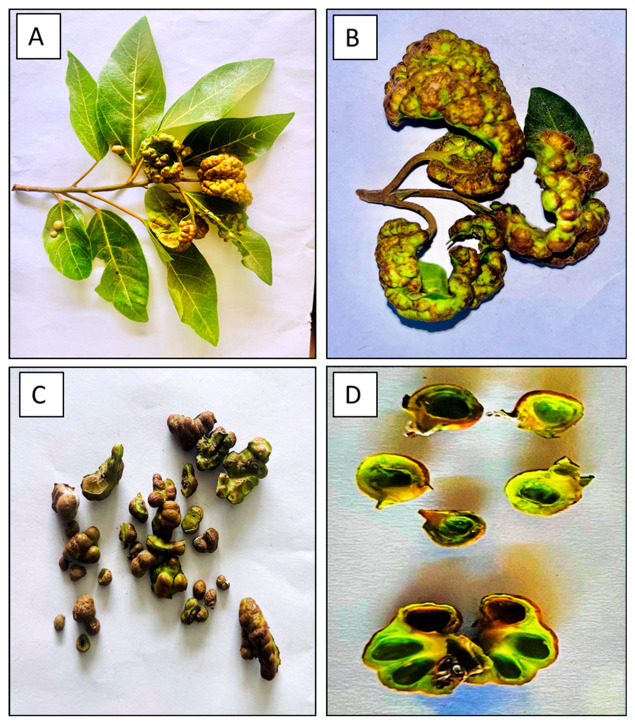
(**A**,**B**) *Ficus recemosa* leaf with gall; (**C**) Isolated galls; (**D**) leaf galls opened.

Gall extracts were obtained from *Ficus recemosa* plants located in Kuttippuram, Malappuram, Kerala, India (coordinates: 10.8456° N, 76.0337° E). Both mature and newly formed galls were collected, with approximately 10 g/10 mL of gall tissue being stored in sterile plastic tubes as global dry content (Figure 1). The samples were brought to the laboratory, washed with distilled water to eliminate external contaminants, and homogenized using a sterile mortar and pestle. Distilled water used as the solvent for extract preparation. The homogenate was then filtered through Whatman No. 1 filter paper and centrifuged at 10,000 rpm for 15 min. The supernatant was collected and refrigerated at 4 °C until it was used for nanoparticle synthesis.

### 2.2. Synthesis of Silver Nanoparticles

Silver nanoparticles (AgNPs) were synthesized using the *Ficus recemosa* gall extract. A 0.01 millimolar (mM) silver nitrate (AgNO_3_) solution was prepared with deionized water. Equal volumes (5 mL each) of the gall extract and the AgNO_3_ solution were mixed in a glass container. The mixture was heated in a standard household microwave oven (model LG-MS-2029 UW, Seoul, Republic of Korea) at 350 watts for 6 min. The formation of AgNPs (0.01 mM) was indicated by the solution turning deep brown. The AgNPs were then purified by placing the mixture in a dialysis bag (molecular weight cut-off: 12,000 Da) and dialyzing against distilled water for 72 h to remove unreacted ions and by-products. The purified AgNPs were stored at room temperature for further use. The pH of the reaction mixture was 7.54 before the microwave treatment and recorded as 5.42 after the treatment. This change in pH affects the formation and properties of AgNPs.

### 2.3. Nanoparticle Characterization

The characterization of biosynthesized AgNPs was verified through multiple analytical techniques, including UV-Vis spectroscopy, Fourier-transform infrared (FTIR) spectroscopy, high-resolution transmission electron microscopy (HR-TEM), zeta potential analysis, and dynamic light scattering (DLS).

#### 2.3.1. UV-Vis Spectroscopy

UV-Vis spectroscopy was used to confirm the synthesis of AgNPs. The optical absorbance of the nanoparticle solutions was recorded using a PerkinElmer UV/Vis spectrometer (Waltham, MA, USA), identifying the characteristic surface plasmon resonance peak indicative of AgNP formation.

#### 2.3.2. Fourier-Transform Infrared Spectroscopy (FTIR)

FTIR spectroscopy was used to analyze functional group interactions in the biosynthesized AgNPs. Comparative FTIR analyses were performed on the *Ficus recemosa* gall extract, the synthesized AgNPs, and the dialyzed AgNPs. Samples were dried, ground with KBr pellets, and analyzed using a PerkinElmer FTIR spectrometer, with spectra recorded as transmittance percentage against wave number in cm^−1^.

#### 2.3.3. HR-TEM Analysis

The structural and morphological characteristics of silver nanoparticles (AgNPs) synthesized from the leaf galls of *F. racemosa* were analyzed using a JEOL JEM 2100 High-Resolution Transmission Electron Microscope (HRTEM) (Tokyo, Japan). The HRTEM, equipped with a LaB6 electron source, operated at 200 kV and 80 kV, providing a lattice resolution of 0.14 nm and point-to-point resolution of 0.19 nm. Images were captured using a Gatan Orius SC200 CCD camera (2K × 2K resolution, Pleasanton, CA, USA) across various scales (1 μm to 2 nm) to obtain comprehensive structural details. This method enabled high-resolution imaging and detailed analysis of the AgNPs’ morphology and structure.

#### 2.3.4. Zeta Potential Analysis

The surface charge and stability of the synthesized AgNPs were evaluated using zeta potential measurements using HORIBA SZ-100 nanoparticle analyzer (Kyoto, Japan). This analysis provided insights into the surface charge distribution and colloidal stability of the nanoparticles.

#### 2.3.5. Dynamic Light Scattering (DLS) Size Analysis

DLS was employed to determine the size distribution of the synthesized AgNPs. The nanoparticle samples were analyzed using a HORIBA SZ-100 nanoparticle analyzer instrument. This technique provided detailed information on the hydrodynamic diameter and size distribution profile of the AgNPs in solution.

#### 2.3.6. LC-MS Q TOF Analysis of Gall Extract

Using LC-MS Q-TOF analysis, the metabolites found in the methanolic extract of the *F. racemosa* gall were examined. The methanolic extract was first precisely poured into a 25 mL volumetric flask to a volume of 1 mL. To guarantee correct dilution, the extract was then combined with an equivalent volume of methanol. The mixture was further diluted to obtain an acceptable concentration for LC-MS analysis. Before being injected into the LC-MS system, the final sample solution was first cleared of any particles using a 0.22-micron syringe filter. Chromatographic separation was performed using an Agilent 1260 HPLC system (Santa Clara, CA, USA) equipped with a C18 column (3.0 mm × 100 mm, 2.7 microns), maintained at 37 °C. The mobile phase comprised formic acid in water (solvent A) and acetonitrile (solvent B), following a gradient elution profile starting at 95% A and 5% B, changing linearly to 75% A and 25% B from 0 to 5 min, to 50% A and 50% B from 5 to 15 min, to 25% A and 75% B from 15 to 25 min, then to 5% A and 95% B from 25 to 35 min, and finally returning to 95% A and 5% B from 35 to 40 min. The flow rate was set at 0.25 mL/min, with a total runtime of 40 min. Mass spectrometric detection was performed using an Agilent 6200 series TOF/6500 series Q-TOF system operated in positive ion mode under the following conditions: gas temperature of 325 °C, gas flow rate of 6 L/min, capillary voltage of 2000 V, nebulizer pressure of 60 Psi, and fragmentor voltage of 175 V. Data acquisition and analysis were conducted using Agilent’s 6200 series TOF/6500 series Q-TOF 10.1 (48.0) software to identify the chemical constituents in the *F. racemosa* gall extract, followed by qualitative analyses to determine compound profiles and potential biological activities. This methodology enables comprehensive analysis of the methanolic extract, facilitating the identification and characterization of bioactive compounds within the sample.

### 2.4. Antibacterial Activity of AgNPs

The antibacterial activity of the synthesized AgNPs was assessed using the agar well diffusion method against two bacterial strains: *Staphylococcus aureus* (Gram-positive) and *Escherichia coli* (Gram-negative). The bacterial cultures were grown in nutrient broth at 37 °C for 18 h to reach the logarithmic phase. Mueller–Hinton agar plates were prepared by pouring molten agar into sterile Petri dishes and allowing it to solidify. The bacterial cultures were then uniformly spread over the surface of the agar plates using a sterile cotton swab to ensure even distribution. Filter paper discs 6 mm in diameter were aseptically punched into the agar. Each disc was filled with 5 µg of the AgNP solution. As a negative control, 5 µg of the leaf gall extract was used, while a standard antibiotic (tetracycline) was used as a positive control. The plates were incubated at 37 °C for 24 h. After the incubation period, the zones of inhibition around the disc, indicating antibacterial activity, were measured in millimeters using a digital caliper. Each experiment was performed in triplicate to ensure the reliability of the results. The mean and standard deviation of the inhibition zones were calculated and recorded. The data from the antibacterial activity assays were statistically analyzed to evaluate the efficacy of the synthesized AgNPs compared to the leaf gall extract and the positive control. 

### 2.5. Anticancer Assay

The cytotoxicity of gall-mediated silver nanoparticles (AgNPs) synthesized from the leaf gall of *Ficus racemosa* was evaluated using Dalton’s lymphoma ascites (DLA) cells. DLA cells were harvested from the peritoneal cavity of tumor bearing mice, washed three times with normal saline, and suspended in 0.1 mL of viable cell solution. This cell suspension was added to tubes containing various concentrations of the synthesized AgNPs, and the volume was adjusted to 1 mL with phosphate-buffered saline (PBS). The assay mixtures were incubated at 37 °C for 3 h, followed by the addition of 0.1 mL of 1% trypan blue. After 3 min, live and dead cells were counted using a hemocytometer. Cyclophosphamide was used as the positive control, while buffer without nanoparticles served as the negative control. The Trypan blue exclusion assay in DLA cells was employed to determine the cytotoxicity of the gall-mediated AgNPs synthesized from the leaf gall of *F. racemosa*.

### 2.6. Environmental Toxicity Studies

Fresh *Allium cepa* (onion) bulbs were selected for chromosomal aberration studies to assess the potential genotoxic effects of silver nanoparticles (AgNPs) synthesized using the leaf gall extract from the plant *Ficus racemosa.* Equal-sized onion bulbs (2n = 16) were incubated in different concentrations of leaf gall-mediated AgNP solutions: 100 µg, 200 µg, 300 µg, 400 µg, and 500 µg, while control bulbs were incubated in distilled water. Hydrogen peroxide (H_2_O_2_) was used as a positive control, and a solvent without nanoparticles served as the negative control. The bulbs were allowed to grow roots until they reached a length of 2–3 cm, after which the roots were excised and prepared for chromosomal aberration analysis using the squash technique. Root nodules were stained with methylene blue, and 1500 cells from the three best preparations for each treatment were examined under a LEICA ICC50E compound microscope (Wetzlar, Germany).

## 3. Results and Discussion

### 3.1. UV-VIS Spectral Analysis

**Figure 2:**
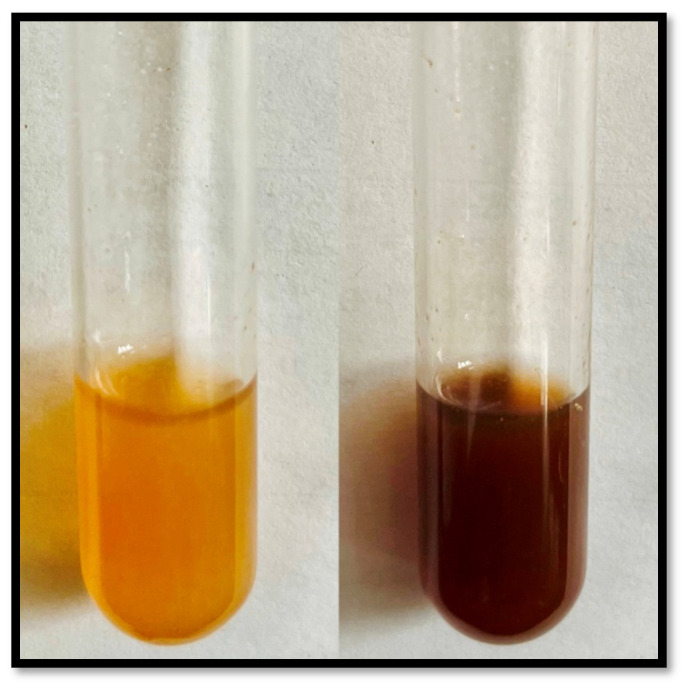
Color change of gall extract mixed with AgNO_3_ and after Microwave treatment; the color change from yellow to brown indicates formation of AgNPs.

**Figure 3:**
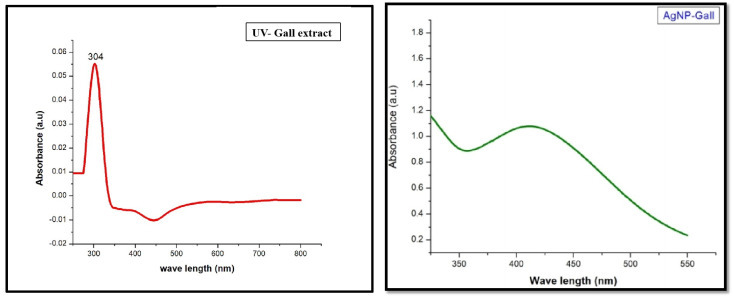
UV-Vis spectra of pure gall extract as reference and synthesised AgNPs.

*Ficus racemosa* exhibited a visual color change from yellow to brown after microwave treatment, indicating the formation of AgNPs (Figure 2). The UV-Vis spectrophotometric analysis of the silver nanoparticles synthesized using the leaf gall extract from the plant showed a distinct surface plasmon resonance (SPR) peak, a key indicator of nanoparticle formation. The absorbance spectrum, as illustrated in the accompanying Figure 3, shows the absorbance of pure gall extract alone (304 nm) and AgNPs synthesised from the gall extract. The absorbance of AgNPs features a significant peak around 425 nm, which is a characteristic signature of AgNPs. The spectrum initiates at approximately 1.2 absorbance units (a.u.) at 350 nm, then decreases steadily, showcasing a notable peak at 425 nm before gradually diminishing at higher wavelengths. This SPR peak not only confirms the successful formation of silver nanoparticles but also demonstrates the efficacy of the leaf gall extract in reducing and stabilizing silver ions. A wide LSPR band may suggest a broad size distribution of silver nanoparticles, as different nanoparticle sizes resonate at varying wavelengths, leading to the band broadening and the absence of additional peaks, indicating the purity of the synthesized nanoparticles [21]. This finding highlights the potential of phytochemicals in the leaf gall extract of *Ficus racemosa* for mediating the green synthesis of silver nanoparticles. Comparable SPR peaks have been observed in other studies involving green-synthesized AgNPs, such as those using *Origanum vulgare* L. extract [22], *Endata rheedii* [23] *Holigarna arnottiana* [11], Brassicaceae seeds [24], and *Malva parviflora* [25]. Additionally, silver nanoparticles synthesized using leaf extracts of *Ocimum sanctum* and its derivative quercetin have shown similar UV peaks [26]. These consistent findings across various plant extracts further validate the effectiveness of green synthesis methods for producing silver nanoparticles.

### 3.2. IR Spectral Analysis

**Figure 4:**
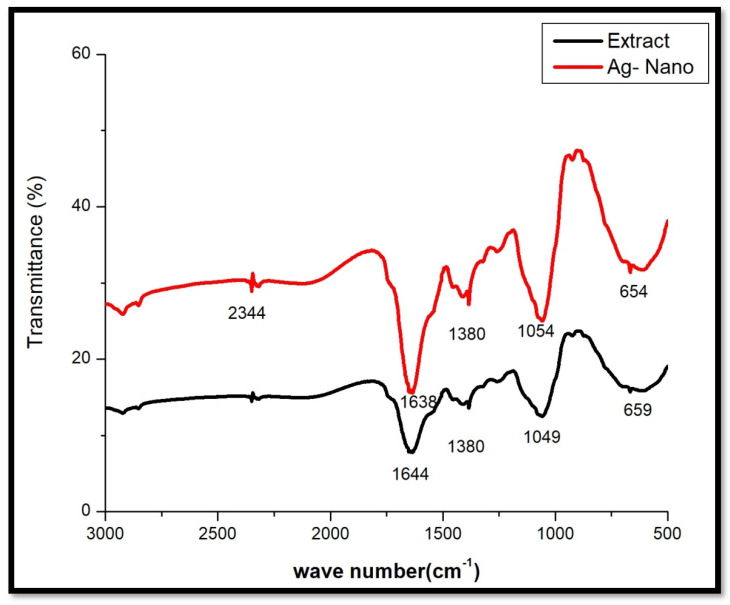
FTIR spectra of lyophilized AgNPs and gall extract.

The FTIR analysis was carried out to identify the functional groups in the lyophilized powder of *Ficus recemosa* gall extract and their role in the synthesis of AgNPs (Figure 4). The gall extract exhibited peaks at 2344 cm^−1^ (C≡C stretching vibrations indicating alkynes) [27], 1638 cm^−1^ (C=C stretching vibrations indicating alkenes or conjugated carbonyl groups) [28], 1380 cm^−1^ (C-H bending vibrations indicating alkanes) [29], 1054 cm^−1^ (C-O stretching vibrations indicating alcohols, ethers, or esters) [30], and 654 cm^−1^ (aromatic out-of-plane bending) [31]. After synthesizing AgNPs, the FTIR spectrum showed peaks at 2345 cm^−1^ [32], 1644 cm^−1^ [33], 1380 cm^−1^ [34], 1049 cm^−1^ [35], and 659 cm^−1^. The observed shifts in peak positions and changes in intensity indicate interactions between silver ions and the functional groups in the gall extract. Specifically, the shifts from 1638 to 1644 cm^−1^ and from 1054 to 1049 cm^−1^ suggest alterations in the environment surrounding the C=C and C-O bonds, likely due to their role in reducing silver ions and stabilizing the AgNPs. The unchanged peak at 1380 cm^−1^ suggests that the C-H bending vibrations remained unaffected. These findings confirm the active involvement of phytochemicals in the gall extract in the synthesis and stabilization of AgNPs, highlighting the efficacy of the green synthesis method.

### 3.3. TEM Analysis Data

**Figure 5:**
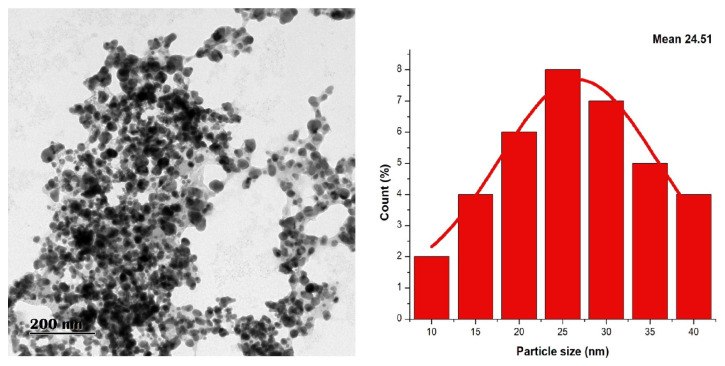
HR-TEM image of AgNPs with histogram showing mean size of nanoparticles.

The transmission electron microscopy (TEM) analysis of silver nanoparticles (AgNPs) synthesized using the leaf gall extract of *F. racemosa* revealed poly-dispersed, spherical nanoparticles with an average size of approximately 20–30 nm (Figure 5). The TEM image and histogram confirmed the varied morphology and size distribution of the nanoparticles, with no significant aggregation observed. The clear and distinct boundaries of the nanoparticles further indicate successful capping by the leaf gall extract, which prevented particle agglomeration. The poly-dispersed size and shape of the silver nanoparticles (AgNPs) synthesized using leaf gall extract, as visualized through TEM, demonstrates the leaf gall extract’s effectiveness in producing stable nanoparticles.

The TEM images reveal that the AgNPs have a mean size of 24.05 nm, with a size distribution ranging from 20 to 30 nm. Correspondingly, DLS analysis indicates a mean hydrodynamic size of 25 nm, falling within the same range. The larger hydrodynamic size compared to the TEM size is due to the inclusion of additional water molecules attached to the nanoparticles.

This characteristic makes them suitable for applications requiring precise nanoparticle attributes. The findings of the current investigation align with previous research [36,37,38,39,40,41]. These studies collectively highlight the potential of various plant extracts in the green synthesis of silver nanoparticles with consistent size and shape.

### 3.4. DLS Analysis of AgNPs

**Figure 6:**
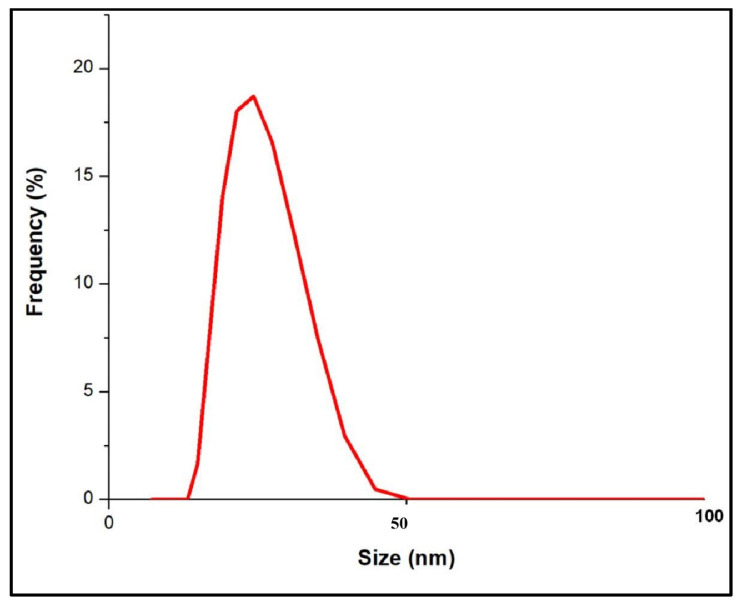
Dynamic Light Scattering of AgNPs.

The Dynamic Light Scattering (DLS) analysis of silver nanoparticles (AgNPs) synthesized using the leaf gall extract of *Ficus racemosa* revealed a polydispersed distribution, predominantly centered around 20–30 nm, and a peak at approximately 25 nm which is slightly higher than the nanoparticle size obtained from TEM (Figure 6). The frequency distribution curve confirms the distribution of the nanoparticle sizes, indicating the effectiveness of the leaf gall extract as a reducing and capping agent. This size distribution suggests minimal aggregation, highlighting the stability of the synthesized nanoparticles. The consistency and size distribution of the AgNPs, as demonstrated by the hydrodynamic size measured through DLS analysis, highlighting the effectiveness of the biosynthesis method using *F. racemosa* leaf gall extract. This method produces stable nanoparticles, making them suitable for various medical, environmental, and technological applications [42]. Similarly, green synthesis using *Azadirachta indica* resulted in AgNPs with a DLS size of 34 nm [43], and synthesis using *Quercus infectoria* gall extract produced AgNPs with a size of 35 nm [44].

### 3.5. Zeta Potential

**Figure 7:**
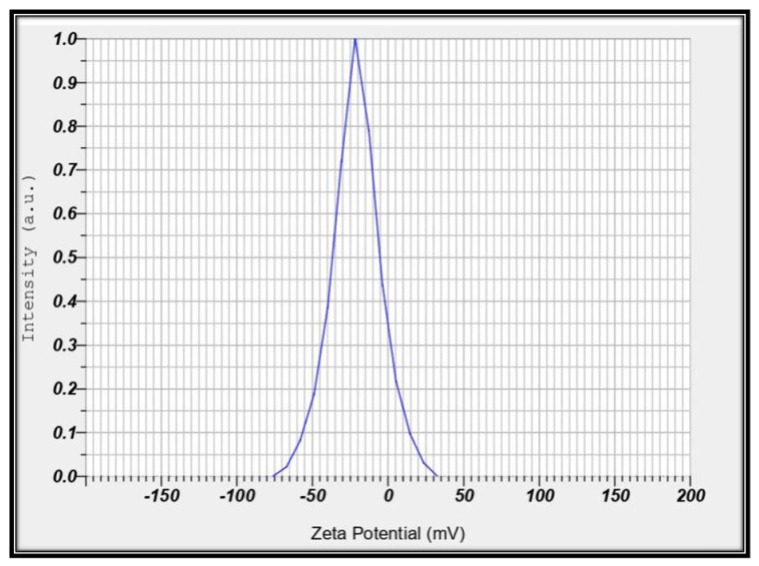
Zeta measurement of AgNPs.

The surface charge and stability of the synthesized AgNPs were assessed using zeta potential measurements, which provided insights into the surface charge distribution and colloidal stability of the nanoparticles [15]. In this study, the zeta potential of AgNPs synthesized from the gall extract was found to be −20.9 mV (Figure 7). This high negative value confirms the repulsion among the particles and indicates that the nanoparticles are stable; the negative sign of the zeta potential is attributed to the compounds in the gall extract, which are responsible for capping and stabilizing the AgNPs. The zeta potential of green-synthesized AgNPs aligns with previous findings. For instance, AgNPs synthesized using an aqueous extract of *Ocimum sanctum* leaf also demonstrated stability [45]. Similarly, AgNPs synthesized using *Phyla dulcis* plant extract maintained a zeta potential between −20 and −24 mV over five weeks, indicating good stability [22]. AgNPs synthesized using *Eucalyptus citriodora* and *Melaleuca cajuputi* exhibited high zeta potential values of −36.49 and −31.16 mV, respectively [46], while those synthesized using *Enicostemma axillare* (Lam.) leaf extract had a zeta potential of −24 mV [47]. These consistent findings highlight the effectiveness of green synthesis methods in producing stable AgNPs.

### 3.6. LC-MS Q-TOF Analysis of Ficus racemosa Gall Extract

**Figure 8:**
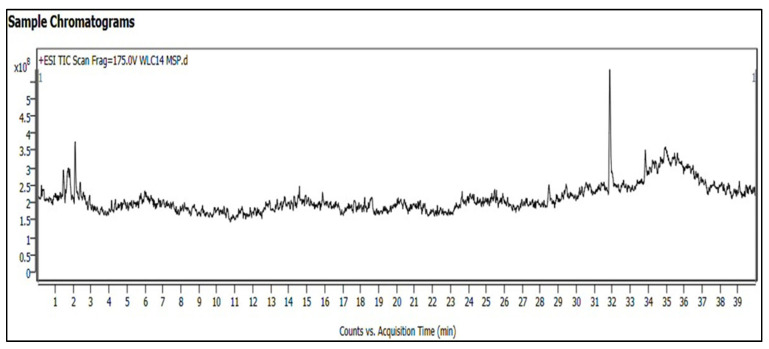
LC-MS Q-TOF analysis of leaf gall extract of *F. recemosa*.

The LC-MS Q-TOF analysis of the methanolic extract from *Ficus racemosa* gall identified a wide range of secondary metabolites (Figure 8). The detected substances belong to various classes, including phenolic compounds, alkaloids, flavonoids, terpenoids, glycosides, and fatty acids and their derivatives. Each class of these compounds contributes uniquely to the extract’s biochemical profile, potentially affecting its biological activities.

(a) Alkaloids

The analysis identified several alkaloids, including Acrimarine H, 1-Piperidinecarboxaldehyde, Sampangine, Istamycin B0, Istamycin AP, and 1-epi-Fortimicin B. Alkaloids are known for their significant pharmacological properties and complex nitrogen-containing structures. Due to their nitrogenous bases, which can donate electrons, these compounds may serve as effective reducing agents.

(b) Flavonoids

Quercetin 4′-glucoside, Quercetagetin 3′-methylether 7-glucoside, 2,3-Dehydrosilybin, Fonsecin, and Glycoperine are among the notable flavonoids that have been found. Flavonoids are potential reducing agents because they have hydroxyl groups that can take part in redox processes. Quercetin, for example, contains many hydroxyl groups that can reduce silver nitrate (AgNO_3_) to produce silver nanoparticles (AgNPs).

(c) Phenolic Compounds

2,4-Dihydroxyacetophenone 5-sulfate, fraxetin, umbelliferone, 4-Aminobenzoic acid, Cinachin Ib, and phenolglucinol are among the phenolic chemicals found in the extract. The hydroxyl groups that are joined to aromatic rings to form phenolics can serve as electron donors in redox processes. Silver ions can be efficiently reduced to metallic silver by these chemicals.

(d) Terpenoids

The terpenoids identified include Cucurbitacin E, 10-Eicosene, 2-Tetradecylcyclobutanone, Turmerone, Nootkatone, Curlone, and Germacrone. These terpenoids can function as reducing agents due to their isoprene units, which contain multiple double bonds, enabling them to participate in redox processes.

(e) Glycosides

The identified glycosides include Hydrocortisone cypionate, Verproside, 2-O-alpha-D-Galactopyranosyl-1-deoxynojirimycin, 3-Phenylpropyl glucosinolate, 4′,8-Dimethylgossypetin 3-glucoside, and 19-Hydroxycinnzeylanol 19-glucoside. These compounds possess sugar moieties with aldehyde or ketone groups, allowing them to act as reducing agents.

(f) Fatty Acids and Derivatives

Phytanic acid, 2-Hexadecanone, (+)-12-Methyl myristic acid, cis-1,2-Dihydroxy-1,2-dihydro-8-methylnaphthalene, Docosanoic acid, 11-Cycloheptylundecanoic acid, Dodecanamide, and Stearamide are among the discovered fatty acids and their derivatives. These substances aid in the reduction of silver ions by having long hydrocarbon chains with functional groups that can contribute electrons.

(g) Other Compounds

Additional compounds identified include Antiarol, Naphthalene dihydrodiol, Sojagol, Sorbaldehyde, Terniflorin, N-Isopropylammelide, Isosorbide, Netilmicin, Pirbuterol, Dyphylline, 2-Hydroxy-6-oxo-7-methylocta-2,4-dienoate, 3-Methoxy-4-hydroxyphenylethylene glycol, and (±)-threo-1-(4-Hydroxyphenyl)-1,2,3-propanetriol. These compounds exhibit various functional groups, such as hydroxyl, carbonyl, and amine groups, which contribute to the reduction process. The secondary metabolites identified in the *F. racemosa* gall extract, including alkaloids (e.g., 1-Piperidinecarboxaldehyde, Sampangine, Acrimarine H), flavonoids (e.g., Quercetin 4′-glucoside, Quercetagetin 3′-methylether 7-glucoside, 2,3-Dehydrosilybin), phenolic compounds (e.g., 2,4-Dihydroxyacetophenone 5-sulfate, Fraxetin, Umbelliferone), terpenoids (e.g., 10-Eicosene, Cucurbitacin E, Solavetivone), glycosides (e.g., 2-O-alpha-D-Galactopyranosyl-1-deoxynojirimycin, Verproside), and fatty acids (e.g., 2-Hexadecanone, (+)-12-Methyl myristic acid, Docosanoic acid), play a pivotal role in the synthesis of silver nanoparticles (AgNPs). These compounds contain various functional groups, such as hydroxyl, carbonyl, and amine groups, which act as reducing agents to convert silver ions (Ag⁺) into metallic silver (Ag⁰). The LCMS results are consistent with the phytochemical and pharmacological profiles of the crude extracts of Ficus racemose [48,49,50,51,52,53]. The functional groups present in flavonoids and phenolic compounds play a crucial role in providing stability to the nanoparticles by preventing aggregation. Additionally, the long hydrocarbon chains in fatty acids and terpenoids contribute to steric stabilization, ensuring the stability and uniform dispersion of AgNPs. This synergistic interaction of secondary metabolites not only facilitates efficient reduction but also enhances the stabilization of AgNPs, highlighting the effectiveness of the green synthesis approach using *F. racemosa* leaf gall extract.

### 3.7. Antibacterial Activity of AgNPS

**Figure 9:**
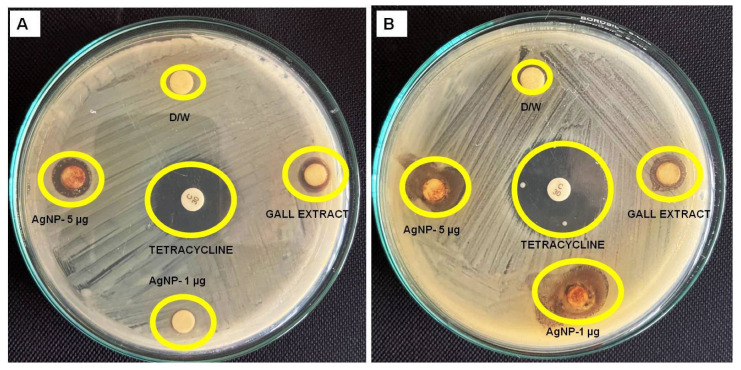
Antibacterial activity of AgNPs evaluated against *Escherichia coli* (**A**) and *Staphylococcus aureus* (**B**).

The antibacterial activity of gall mediated-AgNPs was evaluated against *Staphylococcus aureus* and *Escherichia coli* by measuring the zone of inhibition (Figure 9). The leaf gall extract alone exhibited moderate antibacterial activity, with inhibition zones of 7.33 ± 0.47 mm for *S. aureus* and 6.53 ± 0.47 mm for *E. coli* at a concentration of 5 µg. In contrast, the AgNPs synthesized using the extract showed significantly enhanced antibacterial activity. At 1 µg, the AgNPs produced inhibition zones of 8.66 ± 0.47 mm against *S. aureus* and 7.56 ± 0.47 mm against *E. coli.* Increasing the concentration to 5 µg resulted in inhibition zones of 10.66 ± 0.47 mm for *S. aureus* and 9.66 ± 0.47 mm for *E. coli*. These results clearly illustrate that the synthesized AgNPs are more effective antibacterial agents compared to the extract alone, likely due to their unique physicochemical properties that enhance their ability to disrupt bacterial cell walls and interfere with essential cellular processes. Thus, the AgNPs synthesized with *F. racemosa* leaf gall extract show promise as effective antibacterial agents against both Gram-positive and Gram-negative bacteria, warranting further studies to explore their mechanisms of action and potential applications in medical and environmental settings. Previous research has demonstrated the antibacterial properties of silver nanoparticles, which align with the findings of this study. Silver nanoparticles exert their antibacterial effects by attaching to bacterial membranes, penetrating them, and subsequently disrupting their functionality [54,55,56,57], Previous studies have highlighted the potent antibacterial properties of plant-mediated AgNPs. For instance, silver nanoparticles synthesized using *Mentha piperita* exhibit remarkable antibacterial activity against a variety of bacterial strains [58].

### 3.8. Anticancer Activity

**Figure 10:**
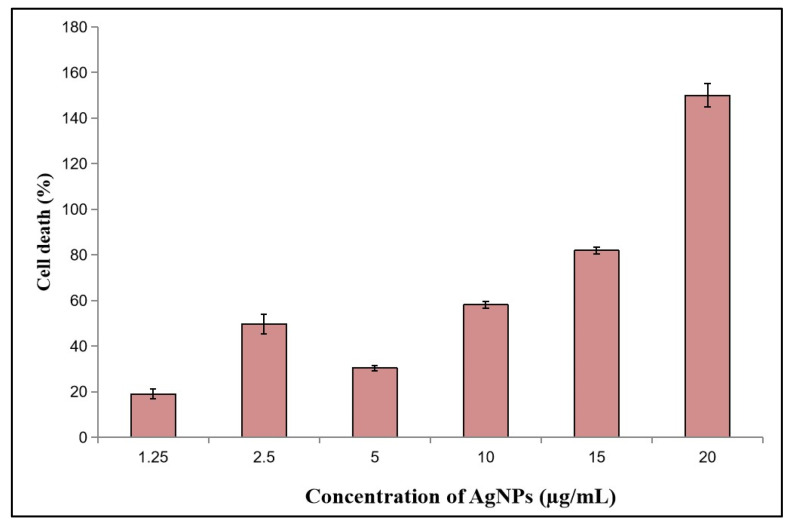
Anticancer activity of AgNPs on DLA cells.

The anticancer activity of silver nanoparticles (AgNPs) synthesized using the methanolic extract of *F. racemosa* gall was evaluated against Dalton’s Lymphoma Ascites (DLA) cells using the trypan blue exclusion method. The results revealed significant cytotoxic effects of the AgNPs on DLA cells (Figure 10). At a concentration of 1.25 µg/mL AgNPs, the cytotoxicity was 19.0 ± 2.2%. As the concentration increased to 2.5 µg/mL, the cytotoxicity rose to 49.7 ± 4.4%. At 5 µg/mL, the cytotoxicity was 30.4 ± 1.2%, and further increased to 58.1 ± 1.6% at 10 µg/mL. A significant cytotoxicity of 81.9 ± 1.6% was observed at 15 µg/mL. At 20 µg/mL, the cytotoxicity reached 150 ± 5%, indicating a high degree of cell death or a possible data recording error as percentages typically do not exceed 100%. The control tube, which contained only the cell suspension, exhibited five dead cells, establishing the baseline for the experiment. These findings indicate that the gall-mediated synthesized AgNPs exhibit potent cytotoxic activity against DLA cells in a dose-dependent manner, demonstrating their potential as effective anticancer agents. Previous studies support the anticancer activity of green-synthesized AgNPs. The potential toxicity of these nanoparticles to public health and the environment has been investigated using methanol leaf extracts of *Gloriosa superba* (L.), *Plumbago indica*, and *Leucaena*. These studies reveal that AgNPs synthesized from these extracts demonstrate significant dose-dependent cytotoxicity against DLA tumor cells, with *Gloriosa superba* (L.) achieving complete cell death at a concentration of 100 µg/mL. This research supports not only the therapeutic potential of these biologically synthesized AgNPs but also the critical need to evaluate their safety and environmental impact [59,60,61].

### 3.9. Chromosomal Aberration Assay

**Figure 11:**
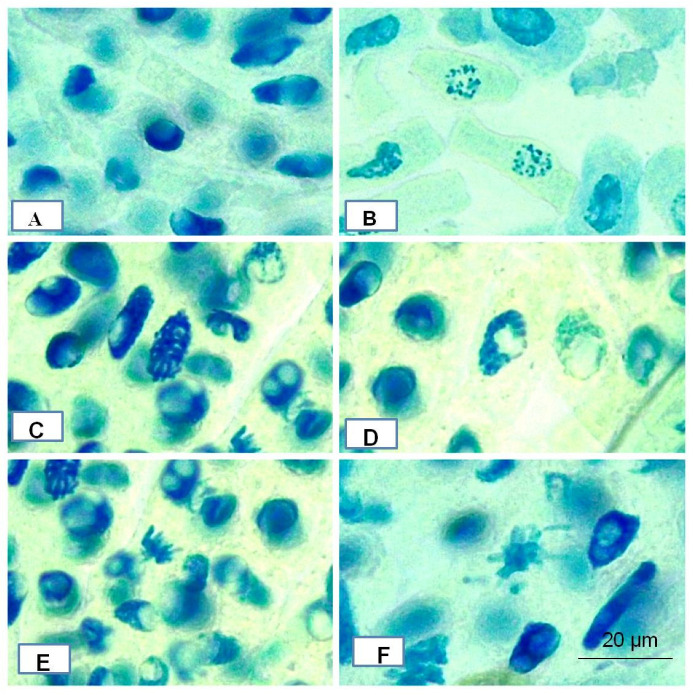
(**A**)—Normal Nuclei, (**B**)—Disintegrating Nuclei, (**C**)—Normal Prophase, (**D**)—Disintegrating Prophase, (**E**)—Normal Metaphase, (**F**)—Sticky Metaphase.

The use of biosynthesized AgNPs in chromosomal aberration studies provided compelling evidence of phytotoxicity induction, with minimal chromosomal abnormalities observed, as illustrated in Figure 11A–F. This was supported by clear findings showing root cells exposed to various concentrations of AgNPs, ranging from 100 µg to 500 µg. The study revealed very minor chromosomal aberrations such as disintegration of nuclei, sticky metaphase, and disintegrating prophase, with the genotoxic potential of AgNPs quantified as a percentage relative to both control and experimental conditions. The data analysis indicated that the Mitotic Index (MI) within the control group remained within the expected range. Similarly, in the experimental group, the percentage of chromosomal abnormalities was comparable to the control, with no significant alterations in %MI (Table 1). These findings suggest that the synthesized AgNPs do not cause substantial genotoxic or mito-depressive effects, indicating their biocompatibility and potential for environmental release without significant pollution to plants. Although previous studies have reported numerous aberrations caused by silver nanoparticles [59,62,63], the present study shows only minor aberrations at lower percentages for green-synthesized AgNPs, compared to earlier results [64,65,66,67,68,69,70].

## 4. Conclusions

In conclusion, this study has successfully demonstrated the environmentally friendly synthesis of silver nanoparticles (AgNPs) using gall extracts from *Ficus racemosa*, leveraging its rich phytochemical composition. The formation of AgNPs was comprehensively validated through UV-Vis spectroscopy, FTIR spectroscopy, TEM imaging, zeta potential analysis, and DLS measurements. UV-Vis spectroscopy confirmed the presence of a distinct surface plasmon resonance peak characteristic of AgNP formation, while FTIR analysis identified specific interactions between silver ions and phytochemicals in the gall extract. TEM images provided visual confirmation of the nanoscale morphology and size of the synthesized particles. Zeta potential and DLS analyses indicated excellent colloidal stability and size distribution of the AgNPs. Biological assays revealed significant antimicrobial activity against *Escherichia coli* and *Staphylococcus aureus*, as demonstrated by the disc diffusion method. Anticancer potential was evidenced through substantial cytotoxicity against Dalton’s Lymphoma Ascites (DLA) cells in the trypan blue exclusion assay. Moreover, the AgNPs exhibited lesser chromosomal aberrations in *Allium cepa* root cells. Overall, this research confirms the efficacy of *Ficus racemosa* gall extracts for green synthesis of AgNPs, offering a sustainable method for producing nanoparticles with robust biological applications. These findings suggest promising applications in medical and agricultural fields, warranting further exploration and application development.

## Figures and Tables

**Table 1:** Effect of AgNPs on *Allium cepa*.

Treatment	Concentration (µL)	Mitotic Index (% ± SD)	Chromosomal Aberration (% ± SD)
Control (Distilled water)	100 µL	11.41 ± 0.21	NIL
	200 µL	12.69 ± 0.21	NIL
	300 µL	13.14 ± 0.21	NIL
	400 µL	14.79 ± 0.21	NIL
	500 µL	15.24 ± 0.21	NIL
Experiment (Biosynthesized AgNPs)	100 µL	11 ± 0.03	NIL
	200 µL	12 ± 0.03	2 ± 0.03
	300 µL	13.8 ± 0.03	3 ± 0.03
	400 µL	14 ± 0.03	5 ± 0.03
	500 µL	15 ± 0.03	6 ± 0.03

## Data Availability

The data generated and analyzed during the current study are available from the corresponding author upon reasonable request.
